# Mapping annual 10-m maize cropland changes in China during 2017–2021

**DOI:** 10.1038/s41597-023-02665-3

**Published:** 2023-11-04

**Authors:** Xingang Li, Ying Qu, Hao Geng, Qi Xin, Jianxi Huang, Shuwen Peng, Liqiang Zhang

**Affiliations:** 1 Beijing Normal University, Faculty of Geographical Science, Beijing, 100875 China; 2 China Agricultural University, College of Land Science and Technology, Beijing, 100083 China

**Keywords:** Agriculture, Geography

## Abstract

China contributed nearly one-fifth of the world maize production over the past few years. Mapping the distributions of maize cropland in China is crucial to ensure global food security. Nonetheless, 10 m maize cropland maps in China are still unavailable, restricting the promotion of sustainable agriculture. In this paper, we collect numerous samples to produce annual 10-m maize cropland maps in China from 2017 to 2021 with a machine learning based classification framework. To overcome the temporal variations of plants, the proposed framework takes Sentinel-2 sequence images as input and utilizes deep neural networks and random forest as classifiers to map maize in a zone-specific way. The generated maps have an overall accuracy (OA) spanning from 0.87 to 0.95 and the maize-cultivated areas estimated by the maps are highly consistent with the records in statistical yearbooks (*R*^2^ varying from 0.83 to 0.95). To the best of our knowledge, this is the first annual 10-m maize maps across China, which largely facilitates the sustainable agriculture development in China dominated by smallholder farmlands.

## Background & Summary

Due to the soaring population and diminishing resources, the world is facing a potential food shortage crisis, where around 70% more food will be needed for human consumption in 2050, as compared to consumption today^1^. The situation could be more urgent in developing countries^2^. Hence, food security is highlighted as a prior sustainable development goal by the United Nation^3^. As one of the top three staple food, maize is cultivated worldwide, with China being one of the top three maize-producing countries. Due to its underdeveloped agriculture management, China, which contributes about 1/5 of the production of global maize, could be an uncertain factor in the global maize trade^4^. Thus, understanding the maize planting pattern of China can be of great significance to facilitate agriculture management and ensure food security.

Recently, GlobeLand30^5^ and FROM-GLC10^6^ have made great progress in land use mapping. However, compared to landcover mapping, the identification of crops is more complex due to the confusing textures and mixed pixels in the coarse-resolution images. By collecting spatial-temporal information of various crops, remote sensing imagery is able to map the changes of crops, which provides an effective tool to simplify agriculture management. For example, to facilitate the management of agriculture, existing mapping produces such as the Cropland Data Layer^7^ (CDL) in the US, and the Agriculture and Agri-Food Canada’s Annual Crop Inventory^8^ (AAFC) have been widely used for crop yield estimation^9^, land use change detection^10^, and crop monitor^11^. Nevertheless, both CDL and AAFC products are derived from Landsat imagery. In certain regions with small cultivation areas, they may struggle to distinguish different crop species accurately. With a 10-meter spatial resolution and a 5-day revisit cycle frequency, Sentinel-2 is able to reduce mixed pixels and provide rich phenological information for different crop types, which has the potential to increase mapping accuracy. Considerable efforts have been made to generate high-resolution maps of various crops. Zheng *et al*.^12^ successfully mapped the distribution of sugarcane in China utilizing long-term Sentinel-2 sequences. Similarly, Pan *et al*.^13^ achieved the identification of double-cropped rice distribution in the Southeast region by discriminating the reflectance differences in Sentinel-1 data. By integrating Landsat and Sentinel-2 imagery, Dong *et al*.^14^ generated high-resolution maps of winter wheat in China.

There are a few works specifically designed for maize mapping with remote sensing data, Shen *et al*.^15^ and Peng *et al*.^16^ utilized time-weighted dynamic time warping to discriminate maize from other agricultural crops, resulting in the production of 30-meter resolution maize maps. With the help of the Sentinel-2B satellite launched in 2017, You *et al*.^17^ produced 10-m maps for maize from 2017 to 2019 in Northeast China with the help of random forest. A similar approach was adopted to map 10-m maize distribution in the Heihe River basin of China^18^ and Makarfi in Northern Nigeria^19^. Wang *et al*. adopted a parcel-based method to extract maize in Jiaozuo (a province of China) by fusing the Sentinel-1 and Sentinel-2 images^19^. However, most of the approaches are applied in small-scale areas, A maize distribution product in China based on a 10-meter spatial resolution has not yet emerged in the current literature due to three potential issues. First, unlike the intensive croplands in developed countries, most agriculture in China is in the form of smallholdings with irregular planting patterns and uncertain farm sizes, which may impede maize mapping^20,21^. Second, the lack of ground truth labels and the interference of noise may decrease the performance of mapping dramatically. Third, due to the spatial-temporal variations, the models trained in one area may perform poorly in the other areas.

To address the above issues, our contributions are three-fold: (1) we collected plenty of samples with ground truth labels across China by field investigation, resolution image interpretation, and the information inferred from statistic yearbooks; (2) To address the problem of spatial and temporal variations, we proposed a recurrent neural network to fully extract temporal features and reduce intra-class variations based on discriminative loss functions; (3) To prevent over-fitting, we adopt zone-specific random forest algorithm in areas with scarce samples. Finally, the maize maps in China at 10-m spatial resolution are obtained for a total of 5 years, from 2017 to 2021. The experimental results indicate that the proposed method is able to generate high-resolution maize maps with reliable accuracy in China dominated by smallholder farmland.

## Methods

### Study area

**Fig. 1:**
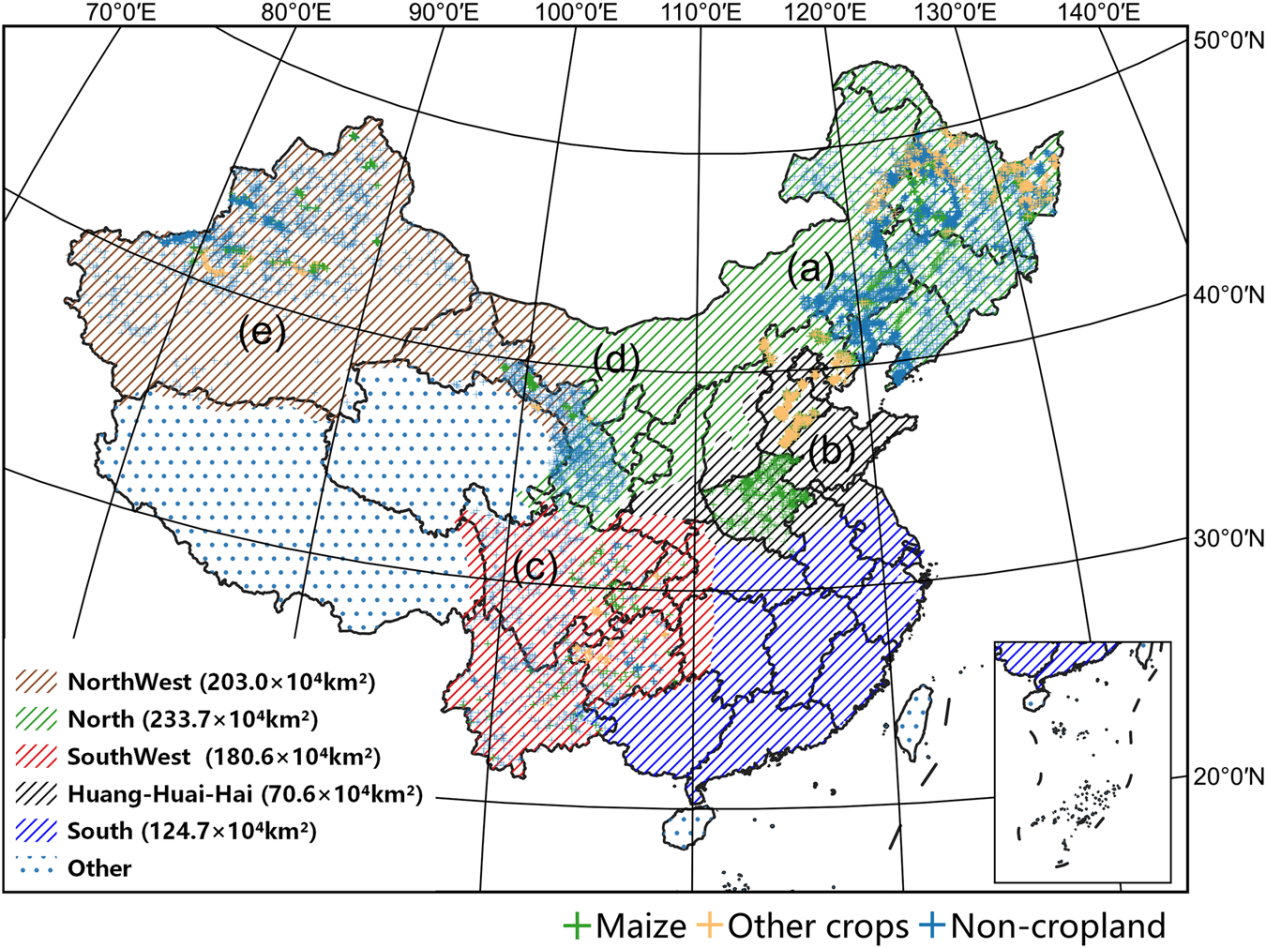
The overview of the collected samples in China. Different textured backgrounds represent different agro-ecological zones, divided into North, Northwest, SouthWest, Huang-Huai-Hai, and South regions. (**a**–**e**) denote the distributions of the samples in North China, Huang-huai-hai, Southwest China, Gansu Province and Xinjiang province, respectively.

The maize-cultivated areas in China are divided into five regions according to local climate and farming practice^22^, i.e., North, Huang-Huai-Hai, Southwest, Northwest and South. According to the China Statistical Yearbook^23–27^, the five study areas cover more than 99.9% of China’s maize planting regions, as shown in Fig. 1.

The climatic conditions in these cultivation zones in China are distinctive. The North China zone experiences a temperate continental climate with cold winters and warm summers. The Huang-Huai-Hai zone has a warm-temperate and semi-humid climate, with distinct seasons and abundant rainfall. The Southwest and Southern China zones have a subtropical monsoon climate with abundant rainfall and high temperatures, while the Northwest region has a temperate continental arid and semi-arid climate with low precipitation and large temperature variations between day and night. Due to natural conditions, five maize-cultivated zones have different cropping systems. For example, maize in the northern regions mainly matures once a year, while in the southern regions, it may mature 2-3 times a year. This inspires us to adopt different models to map maize in different regions.

### The maize mapping procedure

**Fig. 2:**
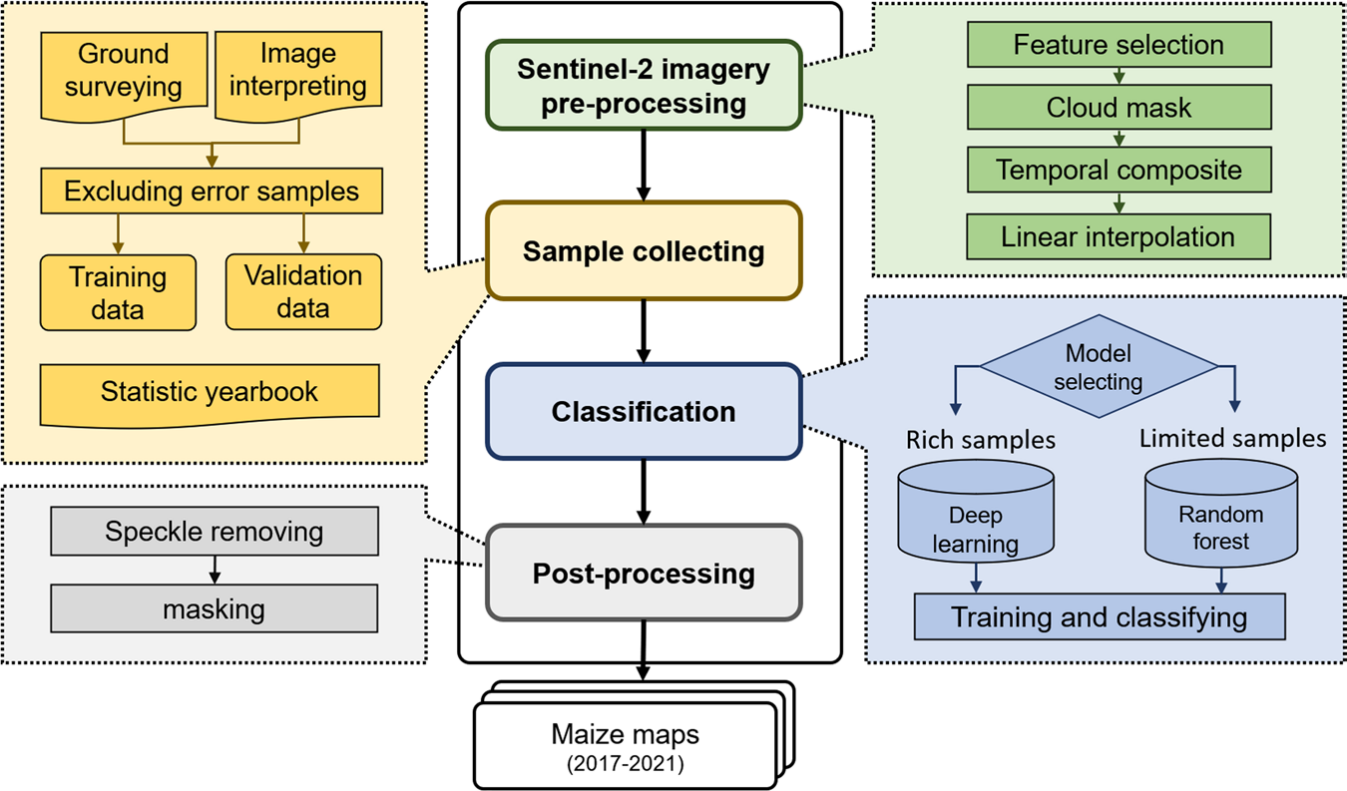
The overview of our maize mapping framework.

The agricultural landscape in China is dominated by smallholder farmlands^4^, where the size of the cropland depends heavily on agroecological and economic environments. In small-scale farmlands, it is hard to identify crop types based on the texture features extracted from images of 10-meter spatial resolution with mixed pixels. Hence, we use the time-series data of Sentinel-2 (S2) imagery as the input to the classification models to extract hierarchical temporal representations. The overview of the framework is shown in Fig. 2, which comprises the following four primary steps, i.e., image pre-processing, sample collecting, classification, and post-processing.

- (1) The image pre-processing step includes band selection, cloud removal, multi-temporal image synthesis, and visual interpolation of samples. More details can be found in the ‘Sentinel-2 images pre-processing’ section.
- (2) In the second step, we collected 79255 ground truth labels from five maize-cultivated zones from 2017 to 2021. Four sample collection methods are described in the ‘Sample collection’ section.
- (3) The third step involves model selection and map generation, where different machine learning models are chosen for different regions, and multi-year maize planting distribution products are obtained. To prevent overfitting, for planting zone (a) with sufficient samples, we propose a deep learning-based model to identify the plants. For plants in other areas (zones b, c, d, e) with limited samples, we categorize plants by adopting the zone-specific random forest model. The maize classification models are introduced in the ‘The classification model’ section.
- (4) The final step is the post-processing procedure, which involves removing speckle points by a circular kernel-based majority filter with a radius of 10 m and masking the maize maps with coarser resolution corn maps. More details can be found in the ‘Post-processing’ section.

### Sentinel-2 image pre-processing

**Fig. 3:**
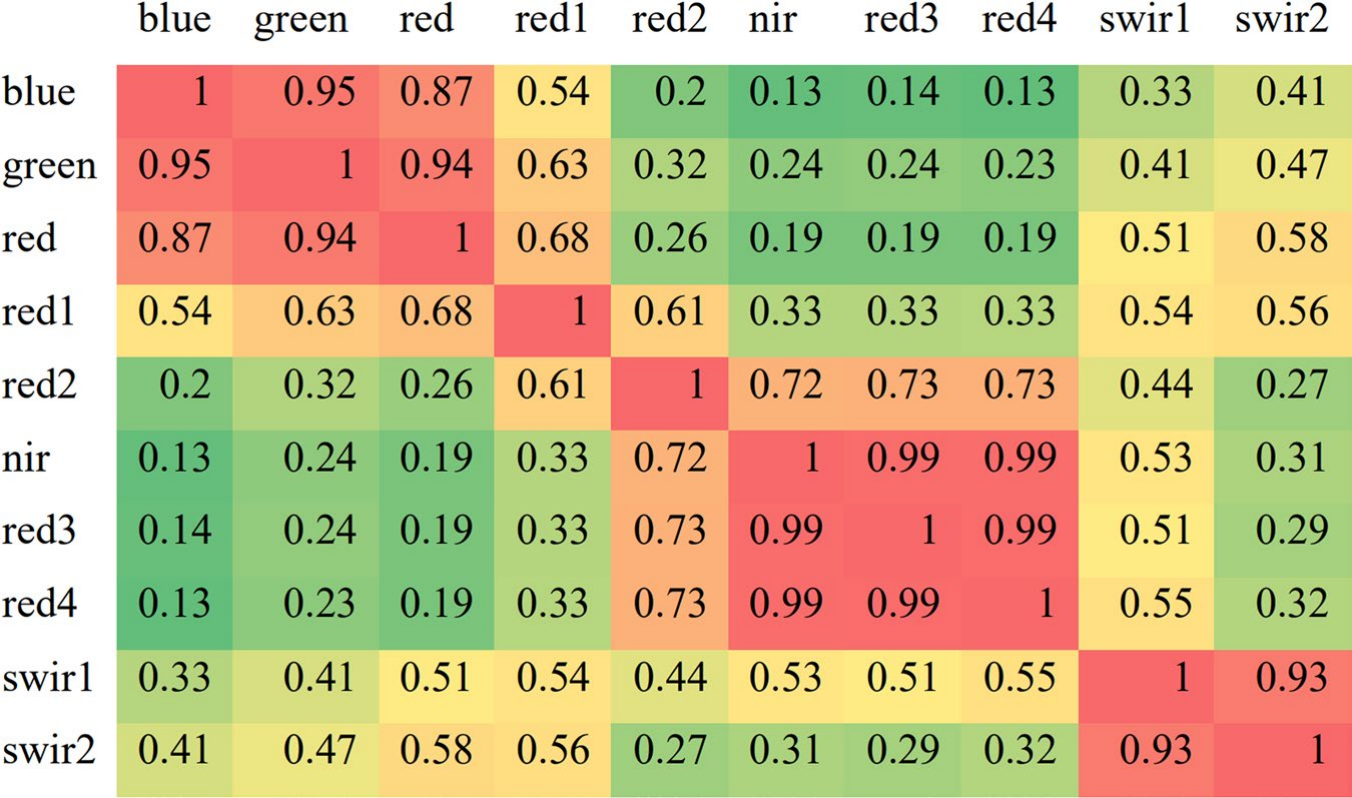
The Pearson correlation analysis of various bands of S2 imagery.

We used the Sentinel-2 top-of-atmosphere (TOA) reflectance images (Level-1C)^28^ acquired from 2017 to 2021 in China as the input for classification tasks. To reduce the spectral redundancy of the images and improve the efficiency of the proposed method, the Pearson correlation coefficients of the different spectral on maize samples are calculated to remove the bands with correlations greater than 0.98, as shown in Fig. 3. The threshold is manually selected used to filter out strongly linearly correlated features. Eventually, 8 S2 image channels, including blue, green, red, red edge1, red edge2, near-infrared (NIR), shortwave-infrared (SWIR)-1 and SWIR-2 channels, were employed for classification. To accelerate model convergence^29,30^, two more channels with widely-used spectral indices, i.e., normalized vegetation difference index (NDVI)^31^ and enhanced vegetation index (EVI)^32^ are stacked with images and adopted as input for maize mapping. The indices are calculated according to Eqs. 1–2

1 $$NDVI=\frac{{\rho }_{nir}-{\rho }_{red}}{{\rho }_{nir}+{\rho }_{red}}$$

2 $$EVI=\frac{2.5\times \left({\rho }_{nir}-{\rho }_{red}\right)}{{\rho }_{nir}+6\times {\rho }_{red}-7.5\times {\rho }_{blue}+1}$$

where ${\rho }_{nir},{\rho }_{red},{\rho }_{blue}$ represent the near infra-red, red, and blue bands of the S2 imagery respectively.

According to the characteristics of the maize phenological^22,33^, we collect images in seven months from April to October (Day of Year (DOY): 90–300) to generate maize mapping. To avoid the accuracy loss caused by cloud contaminations, the adjusted cloud score algorithm^34^ is used to detect and remove clouds. Specifically, the cloud score is the weighted sum of the bright, moist, and snow indices, computed by six bands (aerosols, blue, green, red, nir, and swir). The algorithm^34^ initially detects relatively brighter areas using four spectral bands (blue, aerosols, red, and green) and subsequently calculates a cloud score by taking a weighted sum of two indices, i.e., Normalized Difference Snow Index (NDSI) and Normalized Difference Moisture Index (NDMI), as defined in Eqs. 3, 4. These two indices are computed using three spectral bands (green, red, and shortwave-infrared). To recover the areas covered by cloud, we synthesize S2 images using median values for each 30-day interval. Then the missing pixels are filled by linearly interpolating using images from the preceding and succeeding months.

3 $$NDSI=\frac{{\rho }_{green}-{\rho }_{swir}}{{\rho }_{green}+{\rho }_{swir}}$$

4 $$NDMI=\frac{{\rho }_{red}-{\rho }_{swir}}{{\rho }_{red}+{\rho }_{swir}}$$

where the ${\rho }_{green},{\rho }_{swir},{\rho }_{red}$ denote the green, short-wave infrared and red bands of remote sensing imagery.

### Sample collection

As shown in Fig. 1, the samples are collected from 2017 to 2021 in four study areas with five regions, i.e., (a) North China, (b) Huang-huai-hai, (c) Southwest China, (d) Gansu province and (e) Xinjiang province. Since southern China contributes about 3% of maize planting areas according to the China statistical yearbook, it is hard to collect samples in these areas with on-site surveys Thus, we incorporate samples from the adjacent Southwest China (c) and Huang-Huai-Hai (b) regions to perform classification in southern China. The images are acquired from the Sentinel-2 satellite with samples categorized into three classes, i.e., maize, non-maize and non-cropland, and the number and the distributions of the samples are shown in Tables 1, 2, respectively. The quality of maize samples has a significant impact on the model performance. However, as discussed in Sec. Background and Summary, it is difficult to obtain accurate samples from satellite imagery due to the confusing textures and color features among crop species. Therefore, the samples are collected in the following four ways to guarantee the quality of the samples.

- **(I) The samples collected by field surveys**. We record the positions and species of ground samples by mobile GPS devices. The records of the samples are inspected visually and adjusted manually according to the high-resolution remote sensing imagery.
- **(II) The samples indirectly obtained through the statistical yearbooks of China**. As field surveys are labor-intensive and time-consuming, we generate non-maize samples with the facilitation of the statistical yearbook^23–27^, which contains detailed crop yield records for each county. We assume that the counties without maize-planting records for the past ten years do not have maize-planting farmlands. Then, the collected sample points from the farmlands in these counties are categorized as non-maize.
- **(III) The samples derived from existing products**. To prevent overfitting, we increase the number of samples by gathering maize and non-maize samples from the Xinjiang and Gansu maize mapping products^35^ from local agriculture departments in 2020.
- **(IV) The samples derived from visual interpreting**. The non-cultivated land samples are collected according to the color and texture characteristics of the plants in the multi-phase Sentinel-2 imagery.

**Table 1:** The distributions of the samples in North China.

Class	2017 (a)	2018 (a)	2019 (a)	2020 (a)
Maize(I)	4234	4965	5752	1561
Non-maize (I)	9703	10984	8960	2430
Non-cropland (IV)	3183	5061	7495	465

The alphabet (a) is the maize planting zone in North China, and the Greek numerals indicate different sample collecting methods.

**Table 2:** The distributions of the samples in other maize-planting areas, the alphabets from (b) to (e) represent the maize planting zones of Huang-huai-hai, Southwest China, Gansu province and Xinjiang province, respectively.

Year	2020				2021
Region	(b)	(c)	(d)	(e)	(e)
Maize	2061 (I)	1035 (I)	663 (I)	745(III)	523 (I)
Non-maize	1936 (I)	128(I) 568(II)	678 (II)	0	1119 (I)
Non-cropland	1655(IV)	1134(IV)	856(IV)	1361(IV)	0

### The classification model

#### The proposed deep learning model for maize classification

**Fig. 4:**
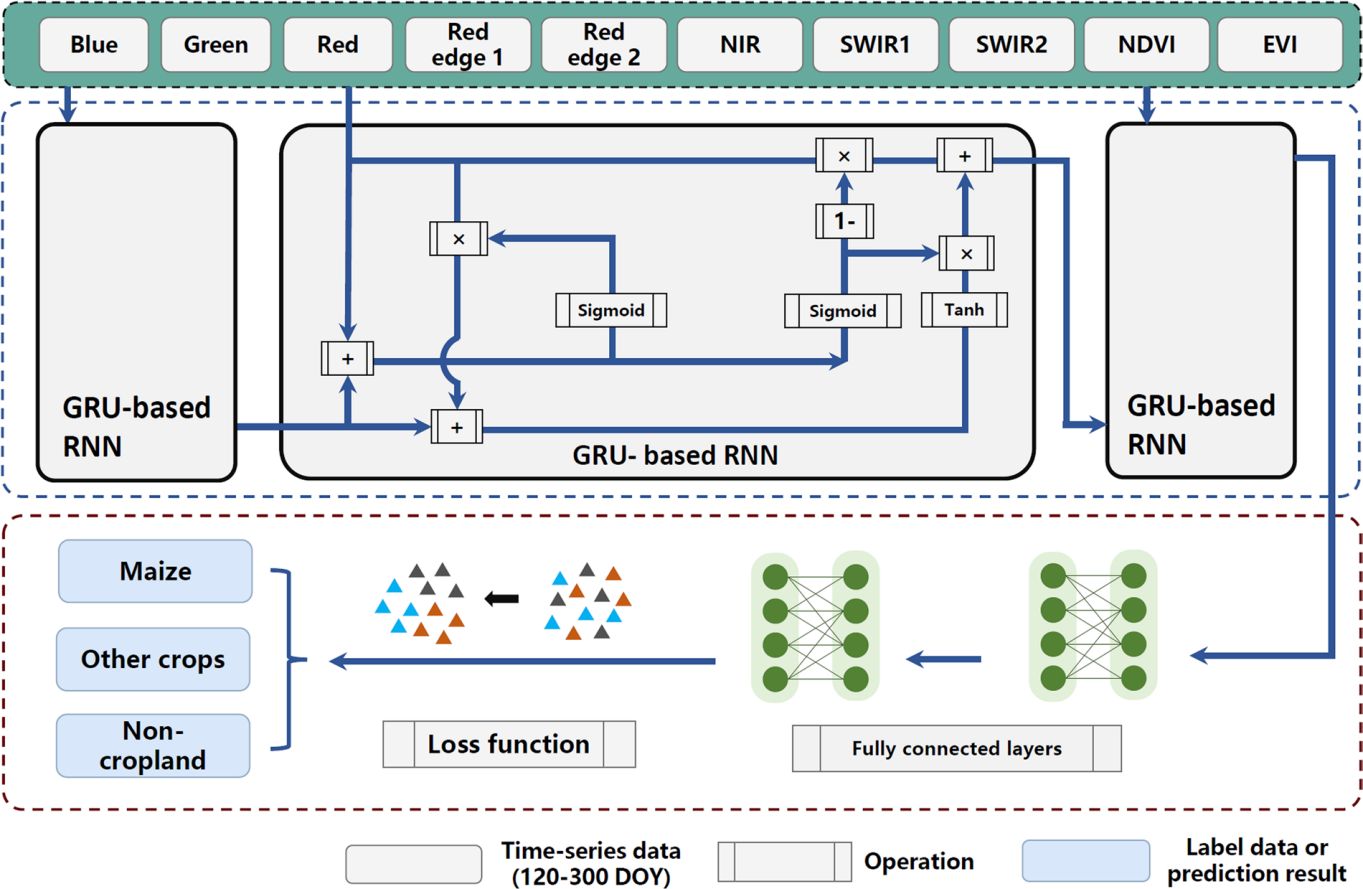
The proposed deep learning model in the maize mapping framework. “ + ” and “×” denote the pointwise addition and multiplication, respectively.

The flowchart of the proposed deep learning-based maize mapping method is shown in Fig. 4. The network architecture mainly consists of two modules, i.e., the feature extraction and classification modules. The input of the network is a sequence of multi-temporal pixels covering the region of interest, which consists of spectral signatures of 8 wavelength bands and the NDVI and EVI indices.

The input data point can be denoted as *I*^*m,s*^ = *I*^4,1^, *I*^4,2^, *I*^4,3^, *I*^4,4^, *I*^4,5^, *I*^4,6^, *I*^4,7^, *I*^4,8^, *I*^4,9^, *I*^4, 10^, where m is the month of the maize growth phenology (from April to October) and s is the spectral index for different bands (Blue, Green, Red, Red Edge1, Red Edge2, NIR, SWIR-1, SWIR-2, NDVI, and EVI).

The feature extractor module consists of a two-layer recurrent neural network (RNN)^36^ to extract temporal information, while the classification module is constructed with two fully connected layers. To be specific, we utilize an RNN with gated recurrent units (GRUs) as the gating mechanism, which has demonstrated superior performance on smaller datasets^37^. The classification module uses a sigmoid activation function at the output layer to predict the class probabilities of maize, other crops, and non-cropland.

The model is optimized using the Adam algorithm^38^. We adopt a cross-entropy loss and a center loss as the objective functions. The cross-entropy loss (*L*_*ce*_) is calculated by comparing the predicted class probabilities with the ground truth labels of the input data, as shown in Eq. 5. To further improve the discriminative power of the learned features, the network also employs the center loss regularization term^39^ (*L*_*center*_) to minimize the distance of the intra-class features (Eq. 6). In other words, it encourages the features to be close to the centers of their respective classes, making the features more discriminative. This is achieved by minimizing the Euclidean distance between the feature vector of each input sample and the center of its corresponding class, as shown in Eq. 7. The combination of cross-entropy loss and center loss allows the model to optimize both the classification accuracy and the feature representation simultaneously, resulting in a more robust and accurate model. The cross-entropy and center losses are defined as:

5 $${L}_{ce}=-\frac{1}{N}\mathop{\sum }\limits_{i=1}^{N}\mathop{\sum }\limits_{j=1}^{C}{y}_{i,j}log\left({\widehat{y}}_{i,j}\right)$$

6 $${L}_{center}=\frac{1}{2}\mathop{\sum }\limits_{i=1}^{N}{\left\Vert f({x}_{i})-{c}_{{y}_{i}}\right\Vert }_{2}^{2}$$

7 $$L={w}_{1}{L}_{ce}+{w}_{2}{L}_{center}$$

where *N* is the number of samples, *C* is the number of classes, *y*_*i, j*_ denotes the ground truth label of the i-th sample for the j-th class, and ${\widehat{y}}_{i,j}$ is the predicted probability of the i-th sample for the *j* class. ${f}_{\left({x}_{i}\right)}$ represents the deep feature of the i-th sample, ${c}_{{y}_{i}}$ represents the center feature of the *y*_*i-th*_ class, and ||·||_2_ is the Euclidean distance. The weights of the two losses, denoted by *w*_1_ and *w*_2_, were set to 1.0 and 0.001, respectively.

To evaluate the proposed method, the collected samples are randomly divided into training, validation, and testing samples with a ratio of 70:10:20. To avoid overfitting, the optimal model with the best overall accuracy on the validation set will be applied to generate maize maps. During the training process, the batch size is set to 50 and the learning rate is 0.001.

#### The zone-specific random forest model

Random forest algorithm^40^ ensembles a mass of decision trees to obtain the most convincing predictions. Compared to deep learning-based model^22^, the random forest model can achieve better performance with less consumption for the classification and regression tasks with limited labels. Hence, we adopt a random forest model to extract maize from remote sensing imagery in the regions outside of Northeast China. To address the problem of spatial variation, we utilized the samples within each region to train the random forest model specific to that region. Then the trained model is used to predict the maize distribution in the given region. The classification task is performed on the Google Earth Engine (GEE) platform^41^, which has been successfully used to perform crop classification^42^, crop yield regression^43^, etc. On the GEE platform, we configured the number of trees to 200 while keeping the remaining parameters at their default values.

### Post-processing

For large-scale and high-resolution maize mapping, the speckle noise is inevitable. The speckle noise typically refers to small, scattered misclassifications or noise present in the generated maps, which is mainly caused by inherent challenges in remote sensing imagery, which can be influenced by atmospheric conditions, sensor noise, and illumination variations. We apply the circular kernel-based majority filter with a 10-meter radius to remove the speckles^42^, which means that the predicted maize patches smaller than 100 square meters are removed. In addition, since predicting all crops in the northwest region with only a few samples is challenging, we introduce the ChinaCropPhen1km maize distribution product^44^ at a spatial resolution of 1 km to facilitate maize mapping. The ChinaCropPhen1km product is utilized as a mask to filter out the pixels incorrectly identified as maize in Northwest China. Specifically, the maize pixels located beyond the 1 km outward buffer of the ChinaCropPhen1km product are filtered out.

## Data Records

Five 10-m maize cropland maps of are generated for planting areas of China from 2017–2021. The data records are shared in the figshare, which is an online open access repository for publishing research data. As the 10-meter resolution product is quite large, we have separated and saved it according to the administrative division codes (adcode). This data set consists of 145 files. The files are named according to the format ‘[adcode]_[year].tif’^45^.

## Technical Validation

The generated maize maps are evaluated from two aspects, i.e., (1) the overall classification accuracy on the testing datasets, and (2) the consistency between the maize-planting areas estimated based on the proposed method and the ones recorded in the statistic yearbooks.

(1) For each maize-cultivated zone, the model with the highest overall accuracy (OA) in the validation set is adopted to predict the labels in the testing set. The sizes of testing set in different zones are 7266(a),1024(b), 910(c), 500(d) and 532(e), respectively. Four matrices, including user accuracy, producer accuracy, overall accuracy, and kappa coefficient^46^, are used to evaluate the accuracy of the generated maps. The evaluation results of the deep learning and random forest models are shown in Table 3. We can observe that the OAs of the five zones vary from 0.83 to 0.95.

**Table 3:** The summary of the model performance in each maize-cultivataed zone.

Region	User accuracy	Producer accuracy	Kappa coefficient	Overall accuracy
North (a)	0.94	0.92	0.89	0.93
Huai-huai-hai (b)	0.85	0.85	0.75	0.83
Southwest (c)	0.96	0.95	0.93	0.95
Gansu (d)	0.94	0.93	0.91	0.94
Xinjiang (e)	0.93	0.93	0.90	0.93

Since the training and testing samples are not acquired in the same year, the performance of the proposed framework may vary in different years due to phenology variations. To validate the robustness of the proposed framework, we conduct experiments in region (a) that owns samples of multiple years. As shown in Table 4, we selected two years from 2017,2018, and 2019, using the samples in one year as training data and the samples in other year as testing data, to validate the performance of the model. The accuracy of the prediction is slightly decreased (on average, the overall accuracy is 0.85), but still maintains a good accuracy, demonstrating the feasibility of the proposed framework.

**Table 4:** The accuracy of classification with training and testing datasets acquired from different years.

Training years	Predicting years	Overall accuracy
2017	2018	0.79
2017	2019	0.86
2018	2017	0.89
2018	2019	0.90
2019	2017	0.82
2019	2018	0.81
		0.85 (average)

**Fig. 5:**
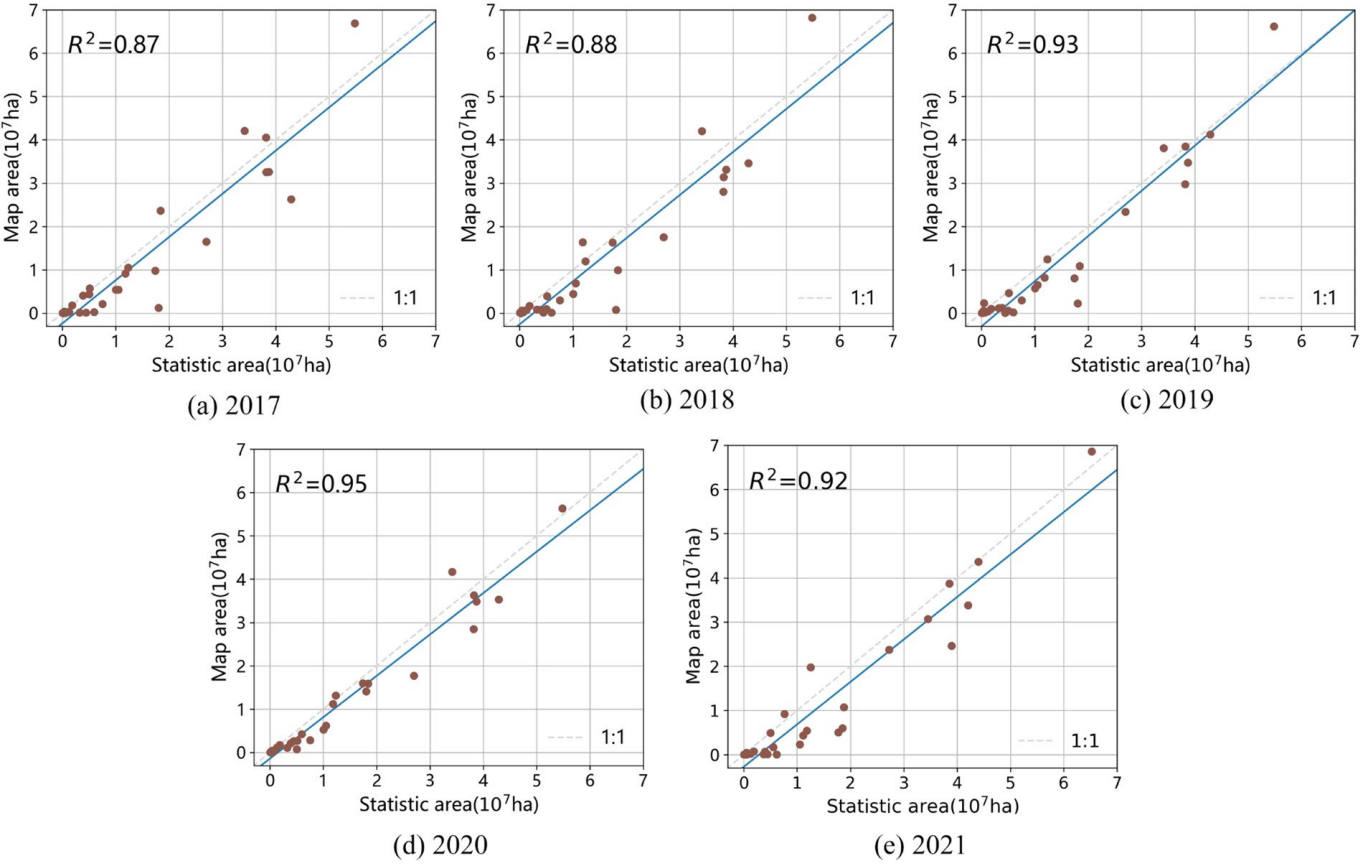
The estimated maize planting area from our annual maize maps with the statistical data at the province level in 2017, 2018, 2019, 2020 and 2021.

**Fig. 6:**
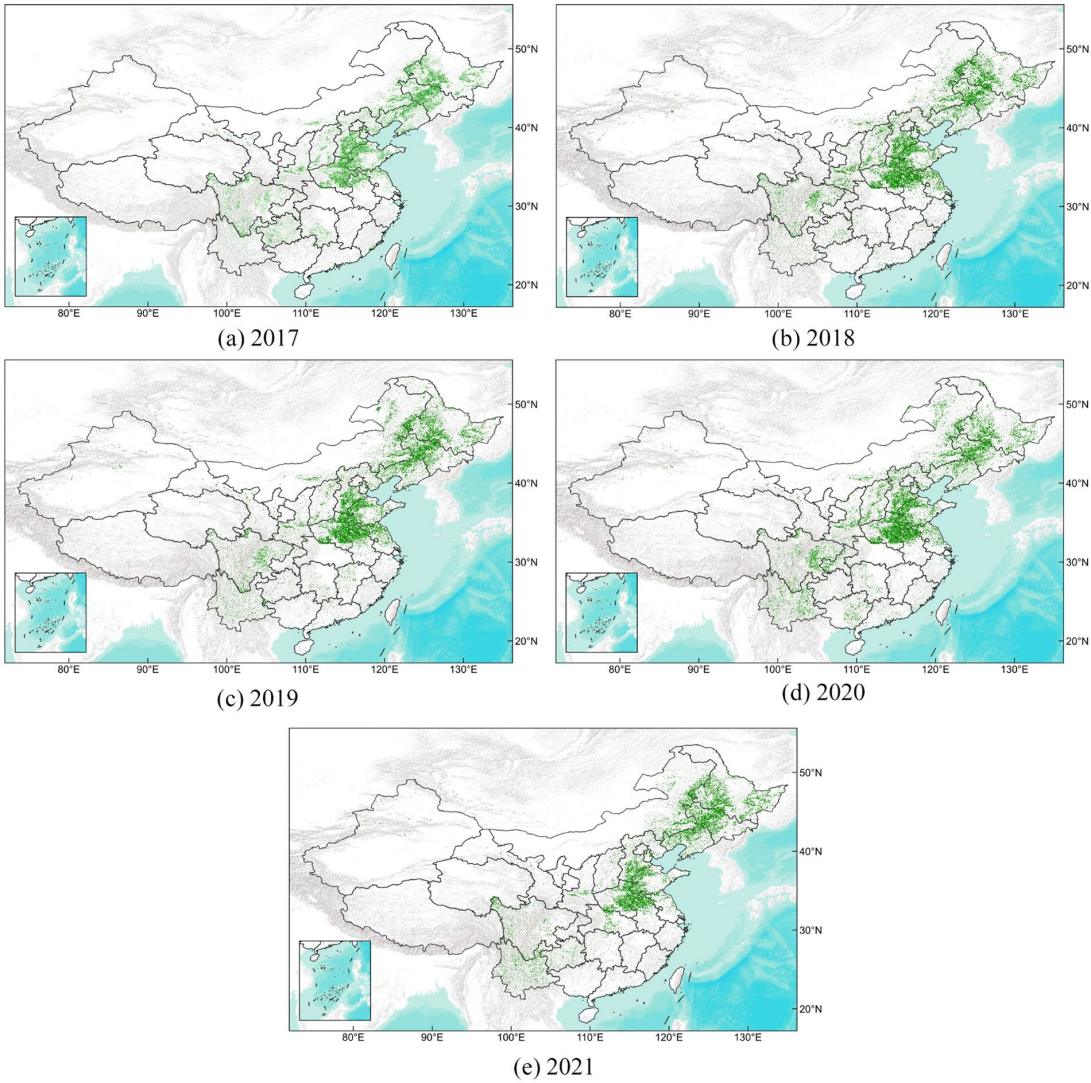
The spatial details of the maize maps from 2017 to 2021.

(2) To further evaluate the proposed framework, the maize-planting areas derived from the annual maize maps are compared with the ones recorded in the statistic yearbooks from 2017 to 2021. Specially, we re-project the annual maps as WGS 1984 Albers for Northern Asia (EPSG: 102025) on the GEE platform to ensure the area units are the same as the yearbook. As shown in Fig. 5, the average coefficient of determination (*R*^2^) is 0.91, with a high value of 0.95 in 2020. These findings indicate that our products are consistent with the statistical yearbook records. The spatial details of the maize maps from 2017 to 2021 is shown in Fig. 6. We can observe that the accuracy is poorer in the provinces located in southern and northwestern China, where there were less number of sample points. The accuracy is higher in Northeast China and Huang-Huai-Hai regions, where there are more sample points. This indicates that the level of uncertainty is mainly caused by the number of sample points, which is a limitation of data-driven models. In the future, we plan to improve our approach by combining data-driven and mechanism-driven models.

## Usage Notes

China is the second-largest maize-producing country in the world. As a developing country dominated by smallholder farmlands and underdeveloped agriculture management, the maize planting areas of China can be easily affected by global climate change^47^, unbalanced economic development^48^, etc. Quantifying the distribution of maize in China is of great significance to global food security. In this paper, we collected 79255 ground truth labels in China and proposed a machine learning based maize classification framework. Then we generate maize maps with a resolution of 10 meter from 2017 to 2021 (Fig. 6). According to the experiments, the maize-planting areas derived from the maize maps are highly consistent with the records in the statistical yearbooks. Therefore, the produced maps can be readily used for agricultural yield estimation and intensive agriculture development. The data is available as binarized spatial raster data, making it simple to be utilized with other products for mathematical and spatial operations. Additionally, our datasets can provide reference and uncertainty analysis for other similar products in Table 5.

**Table 5:** The classification results for each province.

Province	Area(*km*^2^)	2017(*km*^2^)	2018(*km*^2^)	2019(*km*^2^)	2020(*km*^2^)	2021(*km*^2^)
Beijing	16406	387(497)	388(401)	218(337)	393(356)	262(428)
Tianjin	11917	1794(2014)	1688(1868)	1009(1808)	1726(1788)	706(1856)
Hebei	188545	42067(35441)	42003(34377)	38105(34082)	41698(34171)	30715(34541)
Shanxi	156698	9826(18069)	16334(17477)	8070(17150)	15951(17422)	5057(17726)
Neimenggu	1145499	32557(37163)	31390(37421)	38456(37763)	36259(38239)	33779(42046)
Liaoning	148379	16481(26920)	17558(27140)	23409(26750)	17721(26993)	23763(27242)
Jilin	191202	26314(41640)	34633(42315)	41232(42196)	35317(42872)	43616(44012)
Heilongjiang	452538	66894(58628)	68200(63178)	66196(58746)	56361(54806)	68605(65242)
Shanghai	8359	48(30)	105(18)	67(16)	46(12)	7(10)
Jiangsu	106600	1309(5432)	3911(5158)	4647(5042)	2716(5097)	4896(5006)
Zhejiang	105506	47(519)	215(493)	123(764)	235(632)	23(580)
Anhui	140140	3235(11601)	12019(11386)	12473(11965)	13154(12347)	19757(12527)
Fujian	123951	331(268)	556(288)	512(305)	405(330)	227(340)
Jiangxi	166836	220(357)	599(350)	2316(465)	411(476)	385(509)
Shandong	157901	32620(40001)	33118(39347)	34725(38465)	34876(38710)	24608(38970)
Henan	165664	48293(39989)	61487(39190)	60614(38013)	61387(38180)	38733(38533)
Hubei	185937	2149(7948)	2972(7812)	2951(7275)	2811(7519)	9190(7627)
Hunan	211836	4083(3658)	784(3592)	1219(3866)	2140(3843)	696(3976)

The columns 3 to 7 in the table represent the predicted maize planting area for the respective years, while the values inside the parentheses indicate the corresponding statistical area from the yearbook.

## Data Availability

The code utilized for maize mapping is open-source and will be published on GitHub (https://github.com/lixingang/ChinaMaizeCls) and along with the maize maps^45^ (10.6084/m9.figshare.22689751.v17).
